# Low expression of SOD and PRX4 as indicators of poor prognosis and systemic inflammation in colorectal cancer

**DOI:** 10.3389/fonc.2025.1614092

**Published:** 2025-11-04

**Authors:** Sanghyun An, Hye Youn Kwon, Kwangmin Kim, Soo-Ki Kim, Cheol Su Kim, Bora Kim, Hyejin Do, Youngwan Kim

**Affiliations:** ^1^ Department of Surgery, Yonsei University Wonju College of Medicine, Wonju, Republic of Korea; ^2^ Wonju Surgical Research Collaboration, Wonju, Republic of Korea; ^3^ Health Check-up Center, Wonju Severance Christian Hospital, Wonju, Republic of Korea; ^4^ Department of Microbiology, Yonsei University Wonju College of Medicine, South, Wonju, Republic of Korea; ^5^ Department of Internal Medicine, Yonsei University Wonju College of Medicine, Wonju, Republic of Korea; ^6^ Department of Anesthesiology, Chungju Medical Center, Chungju, Republic of Korea; ^7^ Graduate Medical Education, BayCare Health System, Riverview, FL, United States

**Keywords:** colorectal neoplasms, antioxidants, biomarkers, oxidative stress, prognosis

## Abstract

**Objectives:**

Oxidative stress, characterized by an imbalance between the levels of reactive oxygen species (ROS) and antioxidants, plays a critical role in cancer progression. However, the prognostic significance of antioxidant markers in colorectal cancer (CRC) remains unclear. This study aimed to evaluate the expression of antioxidant markers in tumor tissues and investigate their association with clinicopathological features, survival, and systemic inflammation.

**Methods:**

We retrospectively analyzed 70 patients with CRC who underwent curative surgical resection. The tissue levels of superoxide dismutase (SOD), glutathione peroxidase (GPx), peroxiredoxin 4 (PRX4), and thioredoxin (Trx) were measured in freshly frozen tissues, and the patients were classified into high and low expression groups using the 1st quartile as the cutoff. Associations between antioxidant levels in tumor tissue using ELISA and clinicopathological characteristics, laboratory inflammatory markers, and survival outcomes were analyzed.

**Results:**

Low SOD expression was significantly associated with a higher incidence of distant metastases. Similarly, low PRX4 expression was correlated with more aggressive tumor characteristics, including higher rates of distant metastasis, poor differentiation, and advanced T4 stage. Moreover, low PRX4 levels were linked to systemic inflammation, as reflected by increased neutrophil counts and neutrophil-lymphocyte ratio. Although not statistically significant, the low SOD and PRX4 groups exhibited worse 5-year disease-free survival.

**Conclusions:**

Low SOD and PRX4 expression was associated with aggressive tumor features, poor survival, and heightened systemic inflammation in patients with CRC. Given their association with tumor aggressiveness and systemic inflammation, antioxidant markers such as SOD and PRX4 may serve as supportive prognostic biomarkers to help identify patients at risk of adverse clinical outcomes in CRC.

## 1 Introduction

Colorectal cancer (CRC) is the third most commonly diagnosed cancer and a leading cause of cancer-related deaths worldwide (1). Advances in surgical techniques, chemotherapy, radiotherapy, targeted therapy, and immunotherapy have significantly improved treatment outcomes. The development of these treatment strategies has significantly improved the overall survival (OS) of patients with CRC. However, the survival rate for advanced-stage CRC remains poor, with the global OS rate for stage IV metastatic CRC reported to be only 12% (2) and the overall recurrence rate of CRC reported to be 25% (3). These facts highlight the need for further investigation into factors influencing prognosis, disease progression, and novel treatments.

Oxidative stress, which is characterized by an imbalance between the levels of reactive oxygen species (ROS) and antioxidants, has been implicated in cancer development and progression (4–8). Antioxidants, including enzymatic systems such as superoxide dismutase (SOD) and catalase, and non-enzymatic molecules such as glutathione, are essential for neutralizing ROS, preserving cellular redox balance, and maintaining homeostasis (4, 9). Previous studies have suggested that specific antioxidants may be associated with CRC development and progression (10, 11).

Detoxification of ROS operates through a hierarchical system of antioxidant enzymes, each contributing at distinct stages to mitigate oxidative stress. SOD serves as the first line of defense by converting superoxide anions into hydrogen peroxide, which is subsequently neutralized by downstream enzymes such as glutathione peroxidase (GPx) or peroxiredoxins (PRX) to prevent cellular damage. Thioredoxin (Trx) plays a crucial role in this hierarchy by restoring the activity of oxidized antioxidants and ensuring that the system remains functional and efficient. Together, these enzymes maintain the redox balance and influence critical cellular processes, including proliferation, apoptosis, and metabolism, which are pivotal in cancer development (12). Despite their critical roles, comprehensive research investigating antioxidant levels in CRC tissues, particularly in relation to clinicopathological features, hematological markers, and prognosis, remains limited.

Based on these observations, we hypothesized that lower antioxidant levels lead to increased oxidative stress, contributing to more aggressive tumor features in CRC. The aim of this study was to explore the expression of antioxidant markers in fresh surgical specimens from patients with CRC and investigate the association between their expression levels and clinicopathological features, survival, and serological inflammatory markers. These findings can provide insights into their potential as prognostic biomarkers.

## 2 Methods

### 2.1 Population

We enrolled individuals diagnosed with stage I–III CRC according to the American Joint Committee on Cancer (AJCC) guidelines who underwent curative surgical resection between December 2013 and December 2017. To minimize potential sources of bias, we included consecutive patients who underwent curative resection for CRC during the study period and applied consistent inclusion and exclusion criteria. Patients who underwent palliative bypass surgery without radical resection, those with cancers in other organs, and those with a previous history of CRC were excluded. This study was approved by the Institutional Review Board of Wonju Severance Christian Hospital (approval number: CR:319147), and written informed consent was obtained from all participants. This study was conducted in accordance with the ethical principles of the Declaration of Helsinki. Clinical data were analyzed retrospectively using de-identified records; no direct identifiers were accessible to the investigators. The design, conduct, and reporting of this study adhered to the STROBE (Strengthening the Reporting of Observational Studies in Epidemiology) guidelines for observational studies and the REMARK (Reporting Recommendations for Tumor Marker Prognostic Studies) guidelines for biomarker research.

### 2.2 Data collection: clinicopathological data

Patients’ clinical information, including age, sex, medical history, classification recommended by the American Society of Anesthesiologists (ASA), tumor location, tumor markers, pathologic information, and laboratory findings, were obtained from medical records. We defined well-differentiated and moderately differentiated tumors as having favorable differentiation and poorly differentiated and mucinous-type tumors as having poor differentiation. Disease-free survival (DFS) was defined as the period from the date of the index surgery to the date of tumor recurrence or death. OS was defined as the period from the date of index surgery to the date of death. Patient survival data were obtained from the colorectal cancer databases of Wonju Severance Christian Hospital and the Korean National Cancer Center.

### 2.3 Tissue sample preparation

Tumor and normal tissues were harvested and placed in ice-cold RIPA buffer (Pierce Biotechnology Inc., IL, U.S.A.) containing protease inhibitor cocktails (Sigma Chemical Co., St Louis, U.S.A.). The tissues were homogenized at 14000 rpm for 10 min and centrifuged for 5 min, and the supernatant was collected and stored at −80°C until further analysis.

### 2.4 Antioxidant assays

Levels of antioxidants, such as SOD, GPx, PRX, and Trx, in tumor and non-tumor tissues were measured according to the manufacturer’s instructions. The levels of SOD and GPx in colon tumor and non-tumor lysates were measured using colorimetric methods with a Biovision kit (Milpitas, CA, USA), and the Trx level was measured using a fluorescent assay kit (Cayman Chemical Company, Ann Arbor, MI, USA). Protein concentrations were normalized using a Pierce BCA Assay Kit (Thermo Scientific, Rockford, IL, U.S.A.). Each antioxidant assay followed a preparation of standards and reaction processes. The absorbance to determine the concentration was read at the following wavelengths: SOD (450 nm), GPx (340 nm), and Trx (412 nm), using a SpectraMax ABS and ABS Plus Microplate Reader (Molecular Devices LLC, California, United States). An enzyme-linked immunosorbent assay specific for human cytokines was performed to determine the concentrations of peroxidase 4 (PRX4) (AbFrontier, Seoul, South Korea) in colon tumor and non-tumor tissues, according to the manufacturer’s instructions. In brief, the standard stocks were serially diluted, and 100 uL final volumes of standards and samples were added to a 96-well plate. The plates were sealed, incubated, and washed with the washing buffer. The detection antibody was added (100 μL) to each well, and the plate was covered with a new adhesive strip and incubated at room temperature. After incubation and washing, streptavidin-HRP was added to each well (100 μL). Incubation was terminated after 20 min at room temperature, and the plates were kept away from direct light. A substrate solution was added to each well, and color development was terminated using the stop solution. The absorbance was read at 450 nm using a spectrophotometer.

### 2.5 Statistical analysis

We defined the 1st quartile value of each antioxidant marker as the cutoff value and classified patients into high- and low-expression groups. Cases with missing data for specific variables were excluded from the corresponding analyses. Differences in the clinicopathological features between the two groups were analyzed. Categorical variables were analyzed using the chi-square test and presented as frequencies and percentages. The Fisher exact test was performed if the frequency of the data was <5. The normality of all continuous data was tested using the Shapiro–Wilk test. Continuous variables were analyzed using the Student t-test and expressed as mean values and standard deviations. Non-normally distributed data were analyzed using the Mann–Whitney U test and are presented as medians and interquartile ranges. The Wilcoxon signed-rank test was performed to compare antioxidant levels between normal and tumor tissues for each patient. Survival analysis was performed using the Kaplan–Meier curve with a log-rank test. Cox proportional hazards regression model was performed to identify prognostic factors associated with DFS and OS. Variables with a p-value < 0.20 in univariate analysis were included in the multivariate model to adjust for potential confounders. Hazard ratios (HRs) and corresponding 95% confidence intervals (CIs) were calculated for each variable. All statistical analyses were performed using the R software (version 4.2.2; R Foundation for Statistical Computing, Vienna, Austria). Statistical significance was set at p < 0.05.

## 3 Results

### 3.1 Patient characteristics

Seventy patients were included, including 38 (54.3%) men, and the mean age was 69.6 years. The tumor was located in the right colon in 19 patients (27.1%); left colon, 27 (38.6%); and, rectum, 24 patients (34.3%). Fifty-five (78.6%) patients underwent minimally invasive surgery. Stage III and IV disease was observed in 31 (44.3%) and 13 patients (18.6%), respectively. Postoperative chemotherapy was administered to 46 patients (65.7%), whereas radiation therapy was administered to only 1 patient (1.4%). The median follow-up period was 59.5 months. During the follow-up period, tumor recurrence occurred in 17 patients (24.3%), and 5 patients (7.1%) died. Detailed patient characteristics are summarized in **Table 1**.

**Table 1:** Baseline characteristics.

Variable	Number of patients (n=70)	Percentage (%)
Age, mean ± SD	69.6 ± 10.8	
Gender		
Male	38	54.3
Female	32	45.7
Body mass index, mean ± SD	23.4 ± 3.5	
ASA score		
II	35	50.0
III	35	50.0
Medical history		
None	19	27.1
One	18	25.7
Two or more	33	47.1
Tumor location		
Right	19	27.1
Left	27	38.6
Rectum	24	34.3
CEA, median [IQR]	3.1 [2.0; 9.1]	
Operation method		
Open	15	21.4
MIS	55	78.6
T stage		
Tis	1	1.4
3	53	75.7
4	16	22.9
N stage		
0	28	40.0
1	28	40.0
2	14	20.0
M stage		
0	57	81.4
1	13	18.6
TNM stage		
0	1	1.4
2	25	35.7
3	31	44.3
4	13	18.6
Metastatic lymph node, mean ± SD	2.2 ± 3.6	
Harvested lymph node, mean ± SD	24.8 ± 11.1	
Tumor differentiation		
Well-differentiated	13	18.8
Moderately differentiated	53	76.8
Poorly differentiated	1	1.4
Mucinous adenocarcinoma	2	2.9
Tumor size (cm), median [IQR]	4.5 [3.5; 6.0]	
Microsatellite status		
MSS	63	94.0
MSI-H	4	6.0
Chemotherapy		
No	24	34.3
Yes	46	65.7
Radiotherapy		
No	69	98.6
Yes	1	1.4
Recurrence		
No	42	60.0
Yes	17	24.3
Death		
No	45	64.3
Yes	5	7.1

SD, standard deviation; ASA, American Society of Anesthesiologists; CEA, carcinoembryonic antigen; IQR, interquartile range; MIS, minimally invasive surgery.

### 3.2 Antioxidant markers and their relationships with clinicopathologic features

**Figure 1:**
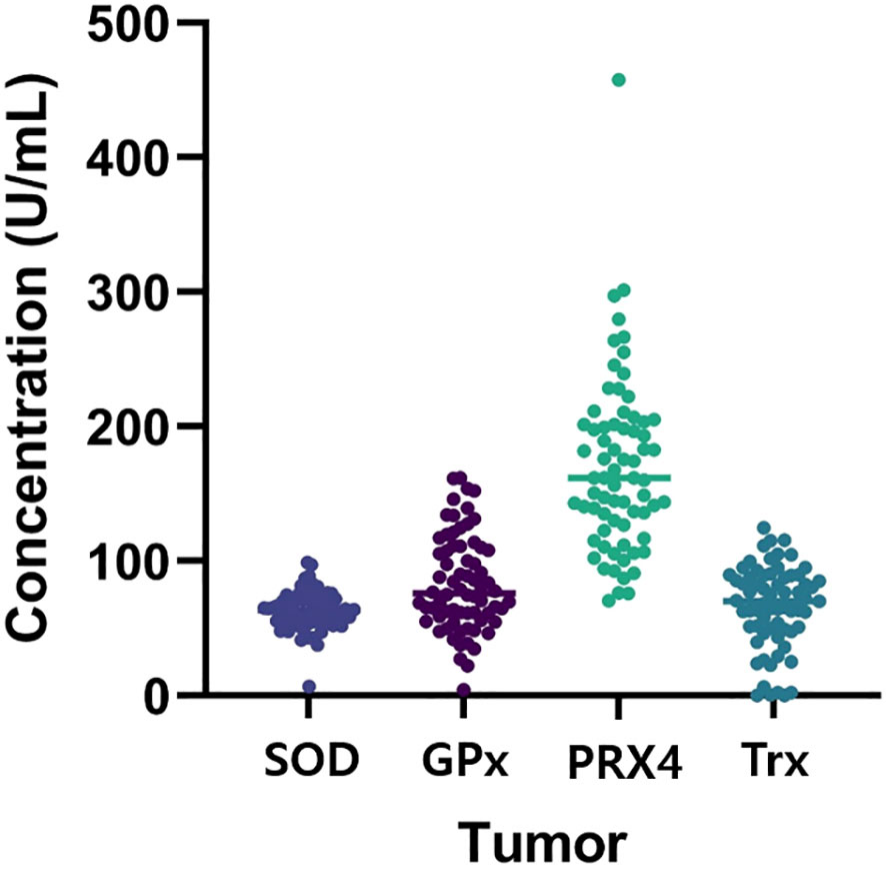
Antioxidant levels in tumor tissue.

**Figure 1** shows the antioxidant levels detected in tumor tissue. The associations between each marker and the clinicopathological features are shown in **Table 2**.

**Table 2:** Correlation of antioxidants with clinicopathologic features.

Variable	SOD			GPx			PRX4			Trx		
	Low (19)	High (51)	p	Low (n=18)	High (52)	p	Low (18)	High (52)	p	Low (17)	High (50)	p
Age	66.1 ± 13.3	71.0 ± 9.5	0.09	69.8 ± 11.6	69.6 ± 10.6	0.95	65.6 ± 14.6	71.1 ± 8.8	0.15	69.5 ± 13.0	69.5 ± 10.2	1.00
Gender			0.24			0.49			1.00			0.53
F	6 (31.6%)	26 (51.0%)		10 (55.6%)	22 (42.3%)		8 (44.4%)	24 (46.2%)		6 (35.3%)	24 (48.0%)	
M	13 (68.4%)	25 (49.0%)		8 (44.4%)	30 (57.7%)		10 (55.6%)	28 (53.8%)		11 (64.7%)	26 (52.0%)	
BMI	22.5 ± 4.0	23.7 ± 3.3	0.19	23.6 ± 3.6	23.3 ± 3.5	0.74	22.8 ± 4.2	23.6 ± 3.3	0.39	24.0 ± 3.8	23.1 ± 3.5	0.37
ASA classification			0.59			0.41			0.41			0.94
II	8 (42.1%)	27 (52.9%)		11 (61.1%)	24 (46.2%)		7 (38.9%)	28 (53.8%)		9 (52.9%)	24 (48.0%)	
III	11 (57.9%)	24 (47.1%)		7 (38.9%)	28 (53.8%)		11 (61.1%)	24 (46.2%)		8 (47.1%)	26 (52.0%)	
Medical History			0.57			0.50			0.32			0.57
none	6 (31.6%)	13 (25.5%)		3 (16.7%)	16 (30.8%)		7 (38.9%)	12 (23.1%)		6 (35.3%)	13 (26.0%)	
one	6 (31.6%)	12 (23.5%)		5 (27.8%)	13 (25.0%)		5 (27.8%)	13 (25.0%)		3 (17.6%)	15 (30.0%)	
≥2	7 (36.8%)	26 (51.0%)		10 (55.6%)	23 (44.2%)		6 (33.3%)	27 (51.9%)		8 (47.1%)	22 (44.0%)	
Tumor Location			0.27			1.00			0.77			0.35
Rt.	3 (15.8%)	16 (31.4%)		5 (27.8%)	14 (26.9%)		6 (33.3%)	13 (25.0%)		4 (23.5%)	15 (30.0%)	
Lt.	10 (52.6%)	17 (33.3%)		7 (38.9%)	20 (38.5%)		6 (33.3%)	21 (40.4%)		5 (29.4%)	21 (42.0%)	
Rectum	6 (31.6%)	18 (35.3%)		6 (33.3%)	18 (34.6%)		6 (33.3%)	18 (34.6%)		8 (47.1%)	14 (28.0%)	
CEA	3.8 [2.0;19.6]	2.7 [2.0; 7.5]	0.52	2.3 [2.0; 4.5]	3.8 [2.0;12.9]	0.13	3.4 [2.0;16.6]	2.9 [2.0; 7.1]	0.68	2.3 [2.0; 4.2]	3.9 [2.0; 9.1]	0.33
T stage			0.49			0.31			0.01			0.05
Tis	0 (0%)	1 (2%)		0 (0%)	1 (1.9%)		0 (0%)	1 (1.9%)		1 (5.9%)	0 (0%)	
3	13 (68.4%)	41 (78.4%)		16 (88.9%)	37 (71.2%)		9 (50.0%)	45 (86.5%)		16 (88.2%)	36 (72.0%)	
4	6 (31.6%)	10 (19.6%)		2 (11.1%)	14 (26.9%)		9 (50.0%)	7 (13.5%)		1 (5.9%)	14 (28.0%)	
N stage			0.95			0.20			0.60			0.54
0	7 (36.8%)	21 (41.2%)		8 (44.4%)	20 (38.5%)		6 (33.3%)	22 (42.3%)		9 (52.9%)	19 (38.0%)	
1	8 (42.1%)	20 (39.2%)		9 (50.0%)	19 (36.5%)		7 (38.9%)	21 (40.4%)		5 (29.4%)	21 (42.0%)	
2	4 (21.1%)	10 (19.6%)		1 (5.6%)	13 (25.0%)		5 (27.8%)	9 (17.3%)		3 (17.6%)	10 (20.0%)	
M stage			0.04			1.00			0.03			1.00
0	12 (63.2%)	45 (88.2%)		15 (83.3%)	42 (80.8%)		11 (61.1%)	46 (88.5%)		14 (82.4%)	40 (80.0%)	
1	7 (36.8%)	6 (11.8%)		3 (16.7%)	10 (19.2%)		7 (38.9%)	6 (11.5%)		3 (17.6%)	10 (20.0%)	
TNM Stage			0.11			0.89			0.08			0.35
0	0 (0.0%)	1 (2.0%)		0 (0.0%)	1 (1.9%)		0 (0.0%)	1 (1.9%)		1 (5.9%)	0 (0.0%)	
2	6 (31.6%)	19 (37.3%)		6 (33.3%)	19 (36.5%)		5 (27.8%)	20 (38.5%)		7 (41.2%)	18 (36.0%)	
3	6 (31.6%)	25 (49.0%)		9 (50.0%)	22 (42.3%)		6 (33.3%)	25 (48.1%)		6 (35.3%)	22 (44.0%)	
4	7 (36.8%)	6 (11.8%)		3 (16.7%)	10 (19.2%)		7 (38.9%)	6 (11.5%)		3 (17.6%)	10 (20.0%)	
Metastatic LNs	1.0 [0.0; 3.0]	1.0 [0.0; 3.0]	0.8	0.5 [0.0; 2.0]	1.0 [0.0; 3.5]	0.41	1.5 [0.0; 4.0]	1.0 [0.0; 3.0]	0.44	0.0 [0.0; 3.0]	1.0 [0.0; 3.0]	0.56
Harvested LNs	24.0 [19.0;29.0]	24.0 [16.5;30.0]	0.71	24.0 [19.0;32.0]	24.0 [16.5;30.0]	0.53	23.5 [19.0;26.0]	25.0 [17.0;31.0]	0.24	21.0 [14.0;26.0]	24.5 [18.0;33.0]	0.09
Differentiation			1.00			1.00			0.02			0.75
Good (wd/md)	18 (94.7%)	48 (96.0%)		17 (94.4%)	49 (96.1%)		15 (83.3%)	51 (100.0%)		16 (100.0%)	47 (94.0%)	
Poor (pd/mucinous)	1 (5.3%)	2 (4.0%)		1 (5.6%)	2 (3.9%)		3 (16.7%)	0 (0.0%)		0 (0.0%)	3 (6.0%)	
Tumor size	5.3 [4.5; 6.1]	4.5 [3.2; 5.6]	0.14	4.5 [3.2; 6.5]	4.5 [4.0; 6.0]	0.85	4.8 [4.5; 6.2]	4.5 [3.1; 6.0]	0.40	4.6 [4.5; 5.5]	4.8 [3.2; 6.0]	0.80
Lymphatic invasion			0.24			0.34			0.21			0.72
0	13 (68.4%)	25 (49.0%)		12 (66.7%)	26 (50.0%)		7 (38.9%)	31 (59.6%)		8 (47.1%)	28 (56.0%)	
1	6 (31.6%)	26 (51.0%)		6 (33.3%)	26 (50.0%)		11 (61.1%)	21 (40.4%)		9 (52.9%)	22 (44.0%)	
Venous invasion			1			0.78			0.12			0.24
0	17 (89.5%)	46 (90.2%)		17 (94.4%)	46 (88.5%)		14 (77.8%)	49 (94.2%)		17 (100.0%)	43 (86.0%)	
1	2 (10.5%)	5 (9.8%)		1 (5.6%)	6 (11.5%)		4 (22.2%)	3 (5.8%)		0 (0.0%)	7 (14.0%)	
Perineural invasion			0.52			1.00			0.83			1.00
0	12 (63.2%)	38 (74.5%)		13 (72.2%)	37 (71.2%)		12 (66.7%)	38 (73.1%)		12 (70.6%)	37 (74.0%)	
1	7 (36.8%)	13 (25.5%)		5 (27.8%)	15 (28.8%)		6 (33.3%)	14 (26.9%)		5 (29.4%)	13 (26.0%)	
Microsatellite status			0.5			1.00			0.08			1.00
MSS	18 (100.0%)	45 (91.8%)		16 (94.1%)	47 (94.0%)		14 (82.4%)	49 (98.0%)		15 (93.8%)	45 (93.8%)	
MSI-H	0 (0.0%)	4 (8.2%)		1 (5.9%)	3 (6.0%)		3 (17.6%)	1 (2.0%)		1 (6.2%)	3 (6.2%)	
KRAS			0.26			0.04			0.49			0.73
Wild	13 (72.2%)	26 (53.1%)		14 (82.4%)	25 (50.0%)		11 (68.8%)	28 (54.9%)		9 (52.9%)	29 (61.7%)	
Mutant	5 (27.8%)	23 (46.9%)		3 (17.6%)	25 (50.0%)		5 (31.2%)	23 (45.1%)		8 (47.1%)	18 (38.3%)	
NRAS			1			1.00			1.00			1.00
Wild	12 (100.0%)	35 (94.6%)		10 (100.0%)	37 (94.9%)		9 (100.0%)	38 (95.0%)		11 (100.0%)	34 (94.4%)	
Mutant	0 (0.0%)	2 (5.4%)		0 (0.0%)	2 (5.1%)		0 (0.0%)	2 (5.0%)		0 (0.0%)	2 (5.6%)	
BRAF			0.66			1.00			1.00			1.00
Wild	18 (100.0%)	44 (93.6%)		16 (94.1%)	46 (95.8%)		15 (93.8%)	47 (95.9%)		16 (94.1%)	44 (97.8%)	
Mutant	0 (0.0%)	3 (6.4%)		1 (5.9%)	2 (4.2%)		1 (6.2%)	2 (4.1%)		1 (5.9%)	1 (2.2%)	
Chemotherapy			0.58			0.70			1.00			0.16
None	8 (42.1%)	16 (31.4%)		5 (27.8%)	19 (36.5%)		6 (33.3%)	18 (34.6%)		9 (52.9%)	15 (30.0%)	
Done	11 (57.9%)	35 (68.6%)		13 (72.2%)	33 (63.5%)		12 (66.7%)	34 (65.4%)		8 (47.1%)	35 (70.0%)	
Radiotherapy			1			0.58			1.00			1.00
None	19 (100.0%)	50 (98.0%)		17 (94.4%)	52 (100.0%)		18 (100.0%)	51 (98.1%)		17 (100.0%)	49 (98.0%)	
Done	0 (0.0%)	1 (2.0%)		1 (5.6%)	0 (0.0%)		0 (0.0%)	1 (1.9%)		0 (0.0%)	1 (2.0%)	

GPx, glutathione peroxidase; SOD, superoxide dismutase; Trx, thioredoxin; PRX4, peroxiredoxin 4; BMI, body mass index; ASA, American Society of Anesthesiologists; CEA, carcinoembryonic antigen; LNs, lymph node; WD, well-differentiated; MD, moderately differentiated; PD, poorly differentiated; MSS, microsatellite stable; MSI, microsatellite instability; MSI-H, microsatellite instability-high; KRAS, Kirsten rat sarcoma viral oncogene homolog; NRAS, Neuroblastoma RAS viral oncogene homolog; BRAF, B-Raf proto-oncogene, serine/threonine kinase.

#### 3.2.1 SOD

The cutoff value for SOD was 54.81 (U/mL). Accordingly, 51 and 19 patients were classified into the high- and low expression groups, respectively. The baseline demographics did not differ significantly between the groups. Distant metastasis occurred in 36.8% of the low-SOD group compared with 11.8% of the high-SOD group (36.8% vs. 11.8%, p = 0.04).

#### 3.2.2 GPx

The cut-off value for GPx was 65.89 (U/mL). Based on this value, 52 and 18 patients were categorized into the high- and low-expression groups, respectively. The baseline demographics did not differ significantly between the groups. However, KRAS mutations were identified in 50.0% of the high-GPx group compared with 17.6% of the low-GPx group (50.0% vs. 17.6%, p = 0.03).

#### 3.2.3 PRX4

The cutoff value for PRX4 was 127.80 (U/mL). Accordingly, 52 and 18 patients were categorized into the high- and low-expression groups, respectively. The low-PRX4 group had a significantly higher proportion of T4 stage tumors (50.0% vs. 13.5%, p = 0.004), distant metastases (38.9% vs. 11.5%, p = 0.026), and poorly differentiated tumors (16.7% vs. 0%, p = 0.021) compared to high-PRX4 group. These findings indicate that reduced PRX4 expression may reflect a more malignant tumor biology.

#### 3.2.4 Trx

The cutoff value for Trx was 39.08 (U/mL). Among the study population, 50 and 17 patients were in the high-and low-expression groups, respectively. There were no significant associations between Trx expression and the clinicopathological features.

### 3.3 Antioxidant markers and laboratory findings

Among the four antioxidant markers, PRX4 was significantly associated with hematological markers. Neutrophil count and neutrophil-lymphocyte ratio (NLR), both reflective of systemic inflammation, were significantly higher in the low-PRX4 group than those in the high-expression group (neutrophil count: 5.6 vs. 4.3, p = 0.027; NLR: 4.3 vs. 2.6, p = 0.003). No significant differences in laboratory findings were observed for the other antioxidant markers (**Table 3**).

**Table 3:** Correlation of antioxidants with laboratory features.

Variable	SOD			GPx			PRX4			Trx		
	Low (19)	High (51)	p	Low (n=18)	High (52)	p	Low (18)	High (52)	p	Low (17)	High (50)	p
WBC	7.5 [6.0; 9.4]	6.6 [5.7; 8.7]	0.23	7.1 [5.9;10.8]	6.9 [5.6; 9.1]	0.51	7.5 [6.4; 9.4]	6.6 [5.2; 9.1]	0.13	7.6 [6.9;10.1]	6.6 [5.5; 9.1]	0.07
Hb	11.9 [10.1;13.5]	12.4 [10.2;13.6]	0.88	12.6 [10.0;13.3]	12.1 [10.2;13.8]	0.93	11.8 [10.6;12.7]	12.4 [9.9;13.9]	0.66	12.6 [9.7;14.3]	11.8 [10.2;13.3]	0.67
PLT	294.0 [235.0;331.5]	259.0 [209.5;328.0]	0.22	269.5 [204.0;315.0]	259.0 [222.5;331.5]	0.6	253.5 [204.0;318.0]	275.5 [219.0;331.0]	0.43	259.0 [237.0;326.0]	269.5 [215.0;330.0]	0.91
Neutrophil	5.0 [3.9; 7.7]	4.7 [3.1; 6.4]	0.2	5.5 [3.1; 8.1]	4.7 [3.4; 6.0]	0.43	5.6 [4.8; 7.6]	4.3 [3.1; 6.4]	0.03	5.6 [4.6; 7.8]	4.8 [3.3; 6.8]	0.12
Lymphocyte	1.4 [1.2;1.7]	1.6 [1.1;1.9]	0.5	1.5 [1.0;1.9]	1.5 [1.2;1.9]	0.54	1.3 [1.0;1.8]	1.6 [1.3;1.9]	0.17	1.7 [1.3;1.9]	1.4 [1.1;1.8]	0.50
NLR	3.6 [2.5; 5.6]	2.8 [2.2; 4.4]	0.35	3.7 [2.1; 6.2]	2.9 [2.3; 4.3]	0.27	4.3 [3.6; 5.8]	2.6 [2.0; 4.1]	0.01	3.8 [2.6; 5.4]	3.0 [2.2; 4.5]	0.29
PLR	228.3 [153.2;268.9]	160.8 [132.7;269.7]	0.41	166.7 [131.3;335.4]	200.0 [132.7;269.1]	0.80	209.0 [149.4;270.2]	162.7 [128.9;273.6]	0.53	172.5 [140.5;263.8]	209.0 [134.7;278.0]	0.72
CRP	1.2 [0.5; 1.9]	0.4 [0.3; 1.2]	0.1	0.9 [0.3; 1.8]	0.4 [0.3; 1.5]	0.42	0.6 [0.3; 1.7]	0.7 [0.3; 1.7]	0.73	0.6 [0.3; 1.1]	0.7 [0.3; 1.8]	0.84
Albumin	3.9 [3.5;4.2]	3.8 [3.5;4.2]	0.89	3.8 [3.2;4.3]	3.9 [3.5;4.2]	0.69	3.8 [3.7;4.2]	3.9 [3.3;4.3]	0.37	4.2 [3.6;4.3]	3.8 [3.4;4.3]	0.57

GPx, glutathione peroxidase; SOD, superoxide dismutase; Trx, thioredoxin; PRX4, peroxiredoxin 4; WBC, white blood cell; Hb, hemoglobin; PLT, platelets; NLR, neutrophil-lymphocyte ratio; PLR, platelet-lymphocyte ratio; CRP, C-reactive protein.

### 3.4 The relationship between antioxidant markers and long-term prognosis

**Figure 2:**
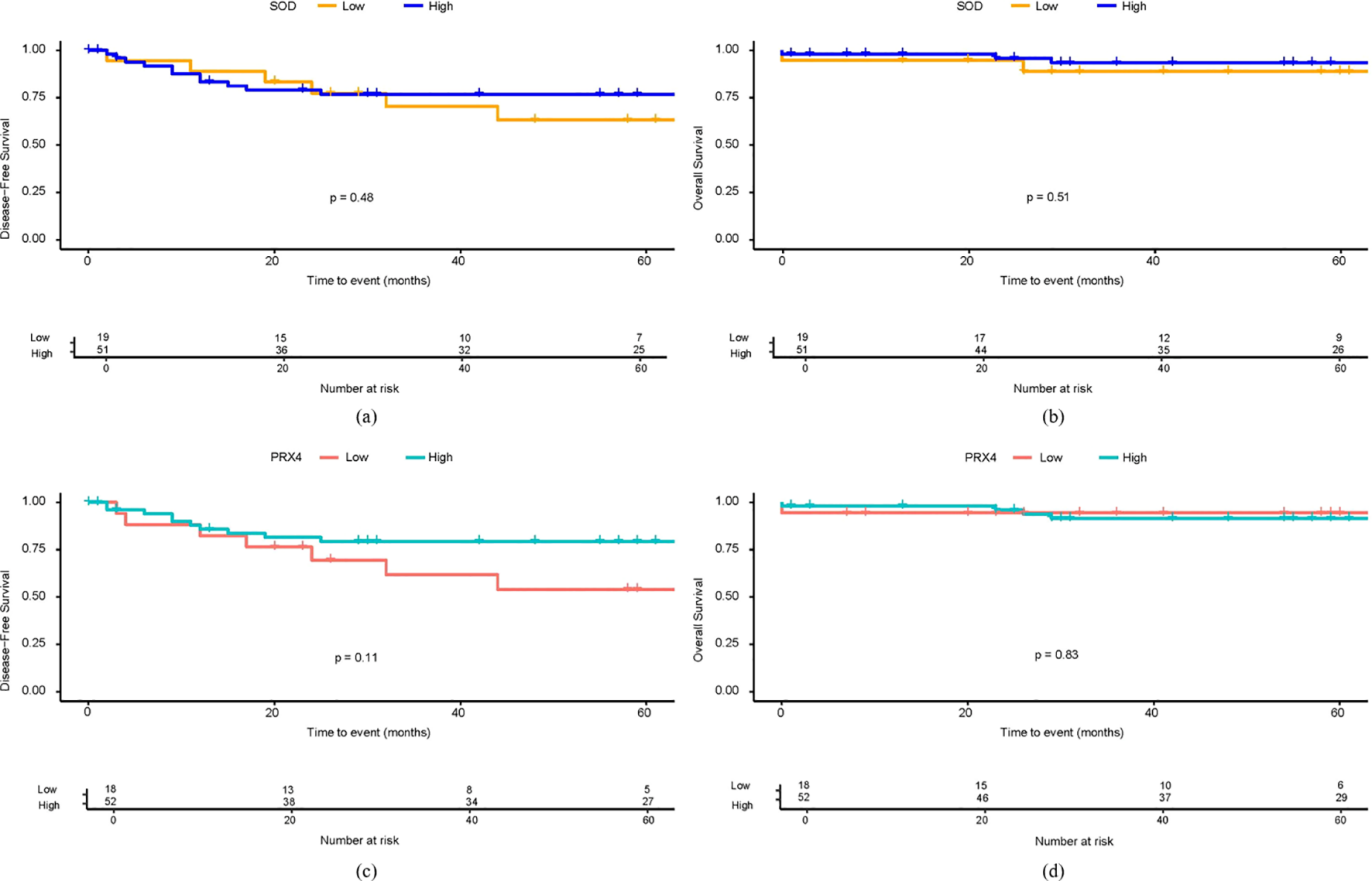
Survival analysis based on SOD and PRX4 expression levels. **(a)** 5-year DFS by SOD expression. **(b)** 5-year OS by SOD expression. **(c)** 5-year DFS by PRX4 expression. **(d)** 5-year OS by PRX4 expression.

In the 5-year DFS analysis of SOD, no significant differences were observed between the high and low expression groups (76.8% vs. 63.3%, p = 0.48). Similarly, no significant differences in the 5-year OS were found between the groups (93.4% vs. 88.8%, p = 0.51). For PRX4, the low expression group showed a poorer 5-year DFS rate than the high expression group (54.1% vs. 79.3%), although this difference was not statistically significant (p = 0.11). Similarly, no significant differences were observed in the 5-year OS rates between the high- and low-PRX4 expression groups (94.4% vs. 91.4%, p = 0.48) (**Figure 2**). In the Cox regression analyses, CEA and poor tumor differentiation were identified as independent predictors of worse DFS (CEA: HR, 2.94; 95% CI, 1.02–8.51; p = 0.046; differentiation: HR, 14.71; 95% CI, 1.75–124.01; p = 0.013) (**Table 4**). For OS, poor differentiation remained the only significant prognostic factor (HR, 24.01; 95% CI, 1.50–384.9; p = 0.025). Antioxidant markers such as SOD and PRX4 showed no independent prognostic impact on DFS or OS in the multivariate models (**Table 5**).

**Table 4:** Cox regression analysis for DFS.

Variable	Category	Univariable HR (95% CI)	P-value	Multivariable HR (95% CI)	P-value
SOD	Low vs. High	0.70 (0.26–1.90)	0.488		
PRX4	Low vs. High	0.46 (0.18–1.21)	0.116	0.82 (0.27-2.46)	0.724
Age		1.02 (0.97–1.08)	0.359		
CEA (ng/mL)	≥5 vs. <5	4.21 (1.55–11.41)	0.005	2.94 (1.02-8.51)	0.046
Stage	III–IV vs. I–II	2.99 (1.47–6.09)	0.003	2.27 (0.62-8.28)	0.213
Differentiation	Poor vs. Well/Moderate	37.07 (5.12–268.44)	<0.001	14.71 (1.75-124-01)	0.013
Adjuvant chemotherapy	Yes vs. No	2.22 (0.64–7.73)	0.21		

HR, hazard ratio; CI, confidence interval; SOD, superoxide dismutase; PRX4, peroxiredoxin 4; CEA, carcinoembryonic antigen.

**Table 5:** Cox regression analysis for OS.

Variable	Category	Univariate HR (95% CI)	P-value	Multivariate HR (95% CI)	P-value
SOD	Low vs. High	0.56 (0.09–3.33)	0.519	–	–
PRX4	Low vs. High	1.26 (0.14–11.30)	0.836	–	–
Age		1.03 (0.93–1.13)	0.6	–	–
CEA (ng/mL)	≥5 vs. <5	1.40 (0.23–8.40)	0.715	–	–
Stage	III–IV vs I–II	2.71 (0.30–24.28)	0.374	–	–
Differentiation	Poor vs Well/Moderate	24.01 (1.50–384.9)	0.025	24.01 (1.50–384.9)	0.025
Adjuvant chemotherapy	Yes vs No	0.68 (0.11–4.07)	0.672	–	–

HR, hazard ratio; CI, confidence interval; SOD, superoxide dismutase; PRX4, peroxiredoxin 4; CEA, carcinoembryonic antigen.

## 4 Discussion

In line with our hypothesis, low antioxidant levels were associated with poor clinicopathological features, survival, and serological inflammatory markers in CRC. Low SOD expression was linked to a higher incidence of distant metastases. Similarly, low PRX4 expression was associated with more aggressive tumor characteristics, including a higher incidence of distant metastasis, poor differentiation, and advanced T4 stage. However, regarding survival outcomes, although Kaplan–Meier curves suggested a trend toward poorer 5-year DFS in the low SOD and PRX4 expression groups, Cox regression analysis did not confirm an independent association between these markers and survival outcomes. Additionally, the low PRX4 group showed elevated neutrophil counts and NLR, indicating a potential link between antioxidant depletion and the inflammatory tumor microenvironment.

SOD, a key enzymatic antioxidant, has been well-documented for its role in neutralizing ROS and maintaining cellular homeostasis. In our study, low SOD expression was associated with a higher rate of distant metastasis, supporting the hypothesis that reduced antioxidant defense contributes to cancer progression. This finding is consistent with previous studies showing that SOD dysregulation promotes oxidative stress, thereby facilitating the metastasis of various cancers. A study analyzing SOD levels in hepatocellular carcinoma tissues reported that reduced SOD expression was associated with older age, larger tumor size, multiple tumor nodules, vascular emboli, poorer OS, and recurrence-free survival (13). In contrast, an observational study by Warsinggih et al. (14) reported that serum SOD levels in patients with CRC were elevated compared to normal reference values. Moreover, higher SOD levels were significantly associated with older age and advanced TNM stages in patients with CRC. Unlike our study, which measured antioxidant levels in tumor tissues, this study assessed antioxidant levels in blood samples. This methodological difference and timing of the measurements may account for these discrepant findings.

PRX4, a unique member of the peroxiredoxin family, is localized in the endoplasmic reticulum and plays a critical role in detoxifying hydrogen peroxide (15). Previous studies have investigated the role of PRX4 expression in tumor progression and the prognoses of various malignancies (15, 16). However, studies on PRX4 expression in CRC remain limited. A study by Yi et al. (17) analyzed PRX4 expression levels in tissue samples from 15 patients with CRC who underwent curative resection and divided the expression levels into four grades. They reported that higher levels of PRX4 expression were associated with unfavorable prognostic factors, including greater depth of invasion, lymph node metastasis, and advanced Dukes stage. In contrast, our study showed that a low PRX4 expression level is associated with aggressive tumor characteristics and increased systemic inflammation. According to the findings of Isohookana et al. (18), a study on patients with pancreatic cancer reported that low expression of peroxiredoxin in tissue samples was associated with unfavorable clinicopathologic features, including larger tumor size, nodal involvement, and poor differentiation. Similarly, another study on patients with hepatocellular carcinoma demonstrated that low PRX4 expression in tissue samples was related to increased tumor growth and invasion and reduced OS, and high PRX4 expression was found to decrease ROS levels in tumor tissue and was associated with better OS (19). However, a dual role of PRX4 was suggested in their study, which revealed that PRX4 knockdown led to a rapid increase in intracellular ROS levels, inducing cell death, and shed light on PRX4’s complex role in cancer progression. Although PRX4’s role is not fully understood, our findings and those of other studies suggest that PRX4 may act as a potential prognostic biomarker and therapeutic target in CRC, reflecting its complex role in tumor progression. Meanwhile, our study demonstrated that low PRX4 expression was associated with elevated inflammatory markers, including neutrophil count and NLR, reflecting an established link between oxidative stress and systemic inflammation (20). Recent research has provided deeper insights into the mechanistic link between low PRX4 expression and systemic inflammation. Specifically, PRX4 deficiency has been shown to potentiate NF-κB signaling and promote the transcription of pro-inflammatory chemokines, thereby facilitating neutrophil infiltration and amplifying inflammatory responses in affected tissues. Large-scale clinical studies further support an inverse association between circulating PRX4 levels and the risk of inflammatory disease, highlighting its potential utility as a prognostic biomarker for systemic inflammation and adverse outcomes. These findings collectively suggest that reduced PRX4 expression may contribute to an inflammatory tumor microenvironment via the NF-κB–neutrophil axis in colorectal cancer (21, 22). However, given that tumor-associated inflammation is often chronic and multifaceted, the lack of association between other antioxidants and inflammatory markers in our study suggests that this relationship may not be directly reflected in all contexts. Further research is required to clarify the intricate interplay between antioxidants and systemic inflammation in patients with CRC.

These conflicting results on the relationship between antioxidants and tumors may be theoretically explained by the dual role of ROS in cancer. Reactive oxygen species are highly reactive molecules derived from oxygen and include superoxide anions (O_2_^-^), hydrogen peroxide (H_2_O_2_), and hydroxyl radicals (OH•). However, excess ROS levels can lead to alterations in the nuclear DNA, inducing mutations and genomic instability that promote cancer initiation. Furthermore, high ROS levels suppress the function of immune cells such as inhibitory T cells and natural killer cells, reducing the ability of the immune system to detect and eliminate early cancerous cells. Conversely, ROS can act as a double-edged sword by contributing to the elimination of cancer cells. Reactive oxygen species can inhibit cancer cell proliferation by suppressing proliferation-signaling pathways, cell cycle progression, and biosynthesis of nucleotides and ATP and by inducing cancer cell death. This is achieved through the activation of stress-related pathways including endoplasmic reticulum stress, mitochondrial apoptotic pathways, p53-dependent apoptosis, and ferroptosis, a form of cell death triggered by iron-dependent lipid peroxidation (23, 24). In a similar context, antioxidants play a dual role in cancer, exhibiting both tumor-suppressive and tumor-promoting effects (25, 26). During the early stages of cancer, antioxidants mitigate ROS-induced DNA damage, protect cells from mutations, and induce genomic instability. However, during tumor progression, elevated antioxidant enzyme expression, as part of the hierarchical interaction of antioxidant systems, enables cancer cells to adapt to elevated ROS levels and promotes survival under oxidative stress. This duality reflects the complex role of antioxidants in cancer. Furthermore, differences in measurement methods and timing may have contributed to variability in the study results, highlighting the need for further research to achieve a more precise understanding of the role of antioxidants in cancer.

Given the critical roles of antioxidant systems in cancer initiation, progression, and therapeutic resistance, there has been growing interest in developing therapeutic approaches that target antioxidants (4, 27, 28). One such example is NOV-002, a glutathione disulfide mimic that modulates redox signaling; it improves response rates in patients with advanced HER2-negative breast cancer when combined with standard chemotherapy (29). Similarly, L-asparaginase, an enzyme that depletes asparagine and indirectly reduces glutathione levels, has demonstrated efficacy in treating acute lymphoblastic leukemia and advanced pancreatic cancer when used in combination with other treatments (30). These examples highlight how targeting the antioxidant system can enhance therapeutic outcomes. However, further studies are required to confirm their broader applicability. On the other hand, a randomized controlled trial of antioxidant supplements, including beta-carotene; vitamins A, C, and E; N-acetyl cysteine; and, selenium, was conducted to evaluate their cancer-preventive effects. A meta-analysis that pooled these studies reported no overall preventive effects of antioxidant supplements on cancer risk (31). Despite these efforts, robust evidence supporting the therapeutic effects of antioxidants is lacking, highlighting the need for further research to clarify their roles and potential in cancer treatment.

This study had several limitations. First, the relatively small sample size may limit the generalizability of the findings, and the retrospective nature of the analysis may have introduced a selection bias. Second, this study was conducted at a single institution, which may limit the generalizability of the findings to a broader population. Third, the study did not include all antioxidant systems, such as other key enzymes, for instance, catalase, which may also play significant roles in cancer progression and redox regulation. Additionally, this study analyzed antioxidant expression at a single time point without considering the dynamic changes in expression levels or temporal trends over the course of disease progression. Finally, this study was designed to examine associations between antioxidant marker expression and clinical prognostic factors rather than to elucidate the underlying biological mechanisms; further mechanistic studies are warranted to clarify these relationships. However, this study has some notable strengths. We utilized a well-defined cohort of patients with CRC who underwent curative resection to ensure consistency in clinical and pathological data. By directly measuring antioxidant markers in tumor and normal tissues, this study provides concrete data on how these markers are associated with cancer progression and prognosis. Furthermore, to our knowledge, this is the first study to analyze tissue-level antioxidant markers in relation to both clinical outcomes and systemic inflammation in patients with CRC.

In summary, the low expression levels of these antioxidants were associated with aggressive clinicopathological features. Furthermore, low antioxidant levels have been linked to high systemic inflammatory status, suggesting that antioxidant depletion may contribute to CRC progression through both tumor aggressiveness and inflammation, underscoring the need for further research to elucidate these mechanisms. These results highlight the potential of SOD and PRX4 as supportive biomarkers for predicting unfavorable clinical features, while further studies are warranted to establish their prognostic utility in CRC.

## Data Availability

The original contributions presented in the study are included in the article/supplementary material. Further inquiries can be directed to the corresponding author/s.
